# Chronic liver disease and cirrhosis increase morbidity in geriatric patients treated surgically for hip fractures: analysis of the US Nationwide Inpatient Sample

**DOI:** 10.1186/s12877-022-02832-y

**Published:** 2022-02-23

**Authors:** Feng-Jen Tseng, Guo-Hau Gou, Sheng-Hao Wang, Jia-Fwu Shyu, Ru-Yu Pan

**Affiliations:** 1 Department of Orthopedics, Hualien Armed Forces General Hospital, Hualien, 971 Taiwan, ROC; 2 Department of Life Science and the Institute of Biotechnology, National Dong Hwa University, Hualien, 974 Taiwan, ROC; 3 Graduate Institute of Medical Sciences, National Defense Medical Center, No. 161, Section 6, Mingchuan E. Road, Neihu District 114, Taipei, 11490 Taiwan, ROC; 4 Department of Orthopaedics, Tri-Service General Hospital, National Defense Medical Center, Taipei, 11490 Taiwan, ROC; 5 Department of Biology and Anatomy, National Defense Medical Center, Taipei, 11490 Taiwan, ROC

**Keywords:** Chronic liver disease, Cirrhosis, Femoral neck fracture, Geriatric, National inpatient sample

## Abstract

**Background:**

This study aimed to evaluate the impact of chronic liver disease and cirrhosis on inpatient outcomes of geriatric hip fracture surgery.

**Materials and methods:**

Using population-based retrospective study design, this study extracted data from the US Nationwide Inpatient Sample (NIS) database 2005–2014, identifying patients aged ≥ 65 years undergoing hip fracture repair. Main outcomes were in-hospital mortality, any/specific complications, non-routine discharge, extended length of stay (LOS) and hospital costs. Associations between cirrhosis, non-cirrhotic chronic liver disease and outcomes were determined using regression analysis.

**Results:**

Data of 347,363 hip fracture patients included 344,035 without liver disease, 1257 with non-cirrhotic chronic liver disease and 2,071 with cirrhosis. After adjustments, non-cirrhotic chronic liver disease was significantly associated with non-routine discharge (OR: 1.247, 95% CI: 1.038–1.498), acute kidney injury (OR: 1.266, 95% CI: 1.039–1.541), extended LOS (OR: 1.285, 95% CI: 1.122–1.473) and hospital costs (beta: 9173.42, 95% CI: 6925.9–11,420.95) compared to no liver disease; while cirrhosis was significantly associated with higher risk of in-hospital mortality (OR: 2.325, 95% CI: 1.849–2.922), any complication (OR: 1.295, 95% CI: 1.143–1.467), acute kidney injury (OR: 1.242, 95% CI: 1.177–1.433), non-routine discharge (OR: 1.650, 95% CI: 1.412–1.928), extended LOS (OR: 1.405, 95% CI: 1.263–1.562) and hospital costs (beta: 6680.24, 95% CI: 4921.53–8438.95) compared to no liver disease.

**Conclusion:**

In geriatric hip fracture patients undergoing surgical repair, non-cirrhotic chronic liver disease and cirrhosis independently predict non-routine discharge, acute kidney injury, prolonged LOS and greater hospital costs, and cirrhosis is also significantly associated with greater risk of any complication and in-hospital mortality.

**Supplementary Information:**

The online version contains supplementary material available at 10.1186/s12877-022-02832-y.

## Introduction

Population aging is increasing life expectancy globally. International reports show an increase in numbers of individuals aged 65 years and over that is shifting the healthcare burden toward age-associated diseases [1]. Together, age-related decreases in bone mass and an increased risk of falls suggest a strong association between age and hip fracture risk [2], and widespread change in the population dynamic will strongly affect the absolute number of fractures [3]. Hip fractures are currently a leading cause of disability and morbidity among older adults [4]. About 30% of older adults with hip fractures die in the following year, others have substantial functional losses, and comorbidities are also increasing [5]. In the United States, an estimated 258,000 geriatric hip fractures were treated in 2010, and anticipated increases in hip fractures as life expectancies increase predict an annual number of hip fractures of 289,000 by 2030 [5]. The annual United States economic burden for managing hip fractures was between $17 and $20 billion in 2010, and hip fracture treatment was ranked 13th among the top 20 most expensive Medicare diagnoses in 2011 [6].

Although many geriatric hip fractures occur as a result of low-energy falls [7], many others arise from underlying low bone density, osteoporosis, or other bone diseases [3]. Certain comorbid chronic diseases are also associated with increased hip fracture risk. In particular, patients with liver cirrhosis, the end-stage of chronic liver disease [8], have both low bone mineral density (BMD) and compromised immune status, which together increase fracture rates and surgical complications [9]. Chronic liver disease is associated with a high risk of osteoporosis and hip fracture through mechanisms ranging from osteoclast-mediated bone resorption to bilirubin-related inhibition of osteoblast proliferation [10–12]. The liver’s role in metabolic processes implicates liver disease as a secondary cause of osteoporosis [10], and a recent meta-analysis showed that cirrhotic patients have an increased risk of fracture [11].

Patients with cirrhosis undergoing elective orthopedic surgeries had increased mortality, LOS and hospital/surgical costs [13], and experienced more complications such as infection, aseptic loosening and need for revision surgeries as reported in total hip and knee arthroplasty [14, 15]. Aside from elective procedures, patients sustaining hip fractures require emergent admission and surgical treatment unless contraindicated by medical instability [16]. Although patients with liver disease have an increased risk of hip fracture as reported by the studies cited above, less is known about the influence of chronic liver disease among comorbidities in geriatric hip fracture repair.

Given the healthcare and economic burdens of both chronic liver disease and hip fracture in the geriatric population, and the fact that few studies have focused specifically on whether and how chronic liver disease may influence the surgical outcomes of geriatric hip fracture patients, a comprehensive investigation of the effects of chronic liver disease on hip fracture repair is warranted. We hypothesized that chronic liver disease increases the risk of postoperative complications, non-routine discharges, prolonged hospital stays and greater costs among geriatric patients undergoing surgical repair for femoral neck fracture. Using a population-based approach, the present study aimed to provide a national snapshot of the impact of chronic liver disease and cirrhosis on inpatient outcomes of repair for geriatric hip fractures.

## Methods

### Study design

This present population-based, retrospective study analyzed data from the US Nationwide Inpatient Sample (NIS) database, which is part of the Healthcare Cost and Utilization Project (HCUP) [17]. The NIS database contains approximately 8 million hospital stays annually derived from about 1,050 hospitals, representing a 20% stratified sample of US community hospitals [17].

### Ethics statement

This study obtained the permission for using the NIS database (2005 to 2014) from HCUP (certificate # HCUP-1L52GWS19), and obeyed the data-use agreement. Because all data in the NIS database are de-identified, the present secondary data using the NIS database does not require the Institutional Review Board approval or informed consent by all subjects.

### Study population

Adults aged 65 years and older, who admitted to hospitals with a principal diagnosis of closed femoral neck fracture, were identified in the NIS database using the International Classification of Diseases, 9th Revision (ICD-9) diagnostic codes (820.00, 820.01, 820.02, 820.03, 820.09, 820.2). Patients undergoing hemiarthroplasty (81.52), total hip arthroplasty (81.51), open reduction and internal fixation (ORIF) (79.35), closed reduction and internal fixation (CRIF) (79.15) or internal fixation alone (78.55) were further identified using primary procedure codes. Patients with elective admissions; open femoral neck fractures; metastatic, nonmetastatic cancer, lymphoma or leukemia; revision hip replacements; or liver transplantation were excluded. Patients lacking complete data for main outcomes and variables were also excluded. The participants were categorized into three groups: no liver disease, non-cirrhotic chronic liver disease and cirrhosis. Supplementary Table 1 displays relevant codes.

### Study outcomes

Study endpoints were 1) in-hospital mortality; 2) any and specific complications; 3) non-routine discharge (defined as discharge to nursing facility, extended care facility, or hospice), 4) extended length of stay (defined as LOS ≥ 75 percentile) and 5) hospital costs.

Postoperative complications were identified by ICD-9 and Clinical Classifications Software (CCS) codes [18] including: postoperative stroke/transient ischemic attack (TIA), delirium, acute myocardial infarction (AMI), pneumonia and respiratory complications, acute kidney injury, hemorrhagic complications, infection/sepsis, neurological complications, thrombocytopenia, digestive system complications, venous thromboembolic complications, and wound/technical complications. Supplementary Table 1 displays relevant codes.

### Covariates

Patients’ demographic and clinical characteristics included age, sex, household income level, race/ethnicity, insurance status (primary payer), fracture type (cervical, peri/intertrochanteric, subtrochanteric), procedure type (hemiarthroplasty, total hip arthroplasty, ORIF, CRIF, internal fixation alone), etiology of cirrhosis and comorbidities. Comorbidities were identified from AHRQ comorbidity measures in the database determined by ICD-9 diagnostic codes using algorithms validated by Elixhauser [19]. Some items were excluded because they were already included as patients’ characteristics. In addition to AHRQ comorbidities, comorbid cardiac arrythmias and tobacco use were also included as covariates.

Hospital-related characteristics (i.e., bed size, location/teaching status, hospital region and costs) and the annual caseload for cervical spine surgery were also extracted from the database as part of the comprehensive data available for all participants.

### Statistical analysis

Categorical variables are presented as count and percentages. Comparisons of proportions between groups for categorical variables were performed using Pearson’s chi-square test or Fisher’s exact test. Continuous variables are presented as mean with standard deviation (SD). Comparisons of continuous variables between groups were performed using ANOVA. Univariate and multivariate regression analysis were conducted to determine associations between cirrhosis, non-cirrhotic liver disease and in-hospital mortality, any complication, acute kidney injury, non-routine discharge, extended LOS and hospital costs. Variables that differed significantly in univariate analysis were included in multivariate regression models after adjustments. A two-tailed *P* value < 0.05 was considered significant. All statistical analyses were performed using SAS 9.4 (SAS Institute, Cary, NC, USA).

## Results

### Study population

Data of 347,363 patients aged 65 years or older who were admitted with closed femoral neck fracture and undergoing either hemiarthroplasty, total hip arthroplasty, ORIF, CRIF or internal fixation alone were extracted from the NIS database 2005–2014.

### Baseline characteristics and outcomes

Patients’ demographic and clinical characteristics and hospital-related information are summarized in Table 1. Among the three patient groups, 344,035 (99.04%) had no liver disease, 1257 (0.4%) had non-cirrhotic liver disease and 2,071 (0.6%) had cirrhosis. Patients’ mean age was 82.86 ± 7.48, 77.11 ± 7.75 and 76.58 ± 7.23 years among those without liver disease, with non-cirrhotic liver disease and with cirrhosis, respectively (*P* < 0.05). The majority of patients were aged 85 years or older (46.35%), female (74.32%), White (88.17%), receiving ORIF (40.84%), with fracture site at peri/intertrochanteric area (58.54%), in the 0–25^th^ percentile of income level (27.23%), and covered by Medicare insurance (91.71%). Significant differences were found between the three groups in age, sex, procedures performed, income level, race/ethnicity, primary payer, comorbidities (i.e., anemia, congestive heart failure, chronic pulmonary disease, coagulopathy, depression, diabetes, hypertension, fluid/electrolyte disorders, other neurological disorders, obesity, peripheral vascular disorders, psychoses, renal failure, weight loss, cardiac dysrhythmias, tobacco use), number of comorbidities, location/teaching status and hospital region (all *P* < 0.05) (Table 1).

**Table 1:** Characteristics of study population by status of chronic liver disease

	Total (*n* = 347,363)	No liver disease (*n* = 344,035)	Non-cirrhotic liver disease (*n* = 1257)	Cirrhosis (*n* = 2071)	*P*-value
**Age**					** < 0.001**
65–74	54,297 (15.63%)	52,900 (15.38%)	527 (41.93%)	870 (42.01%)	
75–84	132,068 (38.02%)	130,737 (38.00%)	471 (37.47%)	860 (41.53%)	
85 +	160,998 (46.35%)	160,398 (46.62%)	259 (20.60%)	341 (16.47%)	
Mean	82.80 (7.51)	82.86 (7.48)	77.11 (7.75)	76.58 (7.23)	** < 0.001**
**Sex**					** < 0.001**
Female	258,120 (74.32%)	256,033 (74.43%)	813 (64.68%)	1274 (61.52%)	
Male	89,193 (25.68%)	87,952 (25.57%)	444 (35.32%)	797 (38.48%)	
**Procedure**					**0.0208**
Hemiarthroplasty	85,388 (24.58%)	84,655 (24.61%)	262 (20.84%)	471 (22.74%)	
THA	8137 (2.34%)	8044 (2.34%)	35 (2.78%)	58 (2.80%)	
ORIF	141,854 (40.84%)	140,468 (40.83%)	528 (42.00%)	858 (41.43%)	
CRIF	85,744 (24.68%)	84,902 (24.68%)	318 (25.30%)	524 (25.30%	
Internal fixation alone	26,240 (7.55%)	25,966 (7.55%)	114 (9.07%)	160 (7.73)	
**Fracture type**					0.0765
Cervical	118,881 (34.22%)	117,810 (34.24%)	411 (32.70%)	660 (31.87%)	
Peri/Intertrochanteric	203,340 (58.54%)	201,334 (58.52%)	742 (59.03%)	1264 (61.03%)	
Subtrochanteric	25,142 (7.24%)	24,891 (7.24%)	104 (8.27%)	147 (7.10%)	
**Etiology of cirrhosis**					**-**
Alcoholic	-	-	-	537 (25.93%)	
Viral	-	-	-	219 (10.57%)	
Other / multiple etiology	-	-	-	1315 (63.50%)	
**Income**					**0.0374**
Lowest quartile	83,140 (24.38%)	82,281 (24.36%)	306 (25.08%)	553 (27.28%)	
Second quartile	92,873 (27.23%)	92,011 (27.24%)	313 (25.66%)	549 (27.08%)	
Third quartile	85,603 (25.10%)	84,794 (25.10%)	310 (25.41%)	499 (24.62%)	
Fourth quartile	79,441 (23.29%)	78,724 (23.30%)	291 (23.85%)	426 (21.02%)	
**Race/ethnicity**					** < 0.001**
White	260,814 (88.17%)	258,433 (88.24%)	893 (79.38%)	1488 (81.94%)	
Black	9569 (3.23%)	9415 (3.21%)	93 (8.27%)	61 (3.36%)	
Hispanic	14,143 (4.78%)	13,864 (4.73%)	81 (7.20%)	198 (10.90%)	
Asian	4250 (1.44%)	4194 (1.43%)	33 (2.93%)	23 (1.27%)	
Others	7047 (2.38%)	6976 (2.38%)	25 (2.22%)	46 (2.53%)	
**Insurance status / Primary Payer**					**0.0009**
Medicare	318,174 (91.71%)	315,173 (91.72%)	1128 (89.88%)	1873 (90.48%)	
Medicaid	3050 (0.88%)	2999 (0.87%)	21 (1.67%)	30 (1.45%)	
Private	20,625 (5.94%)	20,403 (5.94%)	82 (6.53%)	140 (6.76%)	
Self/ no charge /others	5089 (1.47%)	5038 (1.47%)	24 (1.91%)	27 (1.30%)	
**Comorbidities**					
Anemia	104,877 (30.19%)	103,670 (30.13%)	413 (32.86%)	794 (38.34%)	** < 0.001**
Rheumatoid arthritis/collagen vascular diseases	11,490 (3.31%)	11,357 (3.30%)	50 (3.98%)	83 (4.01%)	0.0827
Congestive heart failure	59,564 (17.15%)	58,860 (17.11%)	210 (16.71%)	494 (23.5%)	** < 0.001**
Chronic pulmonary disease	74,658 (21.9%)	73,732 (21.43%)	355 (28.24%)	571 (27.57%)	** < 0.001**
Coagulopathy	22,794 (6.56%)	21,790 (6.33%)	252 (20.05%)	752 (36.31%)	** < 0.001**
Depression	43,560 (12.54%)	43,113 (12.53%)	190 (15.12%)	257 (12.41%)	**0.0218**
Diabetes	74,026 (21.31%)	72,905 (21.19%)	372 (29.59%)	749 (36.17%)	** < 0.001**
Hypertension	244,446 (70.37%)	242,366 (70.45%)	865 (68.81%)	1215 (58.67%)	** < 0.001**
Hypothyroidism	67,041 (19.30%)	66,417 (19.31%)	223 (17.74%)	401 (19.36%)	0.3727
Fluid/electrolyte disorders	100,413 (28.91%)	99,243 (28.85%)	387 (30.79%)	783 (37.81%)	** < 0.001**
Other neurological disorders	62,565 (18.01%)	62,123 (18.06%)	192 (15.27%)	250 (12.07%)	** < 0.001**
Obesity	9506 (2.74%)	9333 (2.71%)	74 (5.89%)	99 (4.78%)	** < 0.001**
Peripheral vascular disorders	24,100 (6.94%)	23,807 (6.92%)	136 (10.82%)	157 (7.58%)	** < 0.001**
Psychoses	11,435 (3.29%)	11,280 (3.28%)	74 (5.89%)	81 (3.91%)	** < 0.001**
Pulmonary circulation disorders	14,865 (4.28%)	14,713 (4.28%)	54 (4.30%)	98 (4.73%)	0.5936
Renal failure	46,342 (13.34%)	45,678 (13.28%)	209 (16.63%)	455 (21.97%)	** < 0.001**
Valvular disease	39,248 (11.30%)	38,861 (11.30%)	128 (10.18%)	259 (12.51%)	0.1014
Weight loss	15,922 (4.58%)	15,646 (4.55%)	95 (7.56%)	181 (8.74%)	** < 0.001**
Cardiac dysrhythmias	92,335 (26.58%)	91,529 (26.60%)	289 (22.99%)	517 (24.96%)	**0.0037**
Tobacco use	52,477 (15.11%)	51,737 (15.04%)	307 (24.42%)	433 (20.91%)	** < 0.001**
**Number of comorbidities:**					** < 0.001**
0	9501 (2.74%)	9453 (2.75%)	24 (1.91%)	24 (1.16%)	
1	33,786 (9.73%)	33,585 (9.76%)	81 (6.44%)	120 (5.79%)	
2	62,573 (18.01%)	62,134 (18.06%)	186 (14.80%)	253 (12.22%)	
≥ 3	24,103 (69.52%)	238,863 (69.43%)	966 (76.85%)	1674 (80.83%)	
**Hospital bed size**					**0.1271**
Large (> 450)	206,113 (59.9%)	204,092 (59.57%)	759 (60.72%)	1262 (61.23%)	
Medium (250–450)	93,441 (27.01%)	92,570 (27.02%)	348 (27.84%)	523 (25.38%)	
Small (< 250)	46,341 (13.40%)	45,922 (13.40%)	143 (11.44%)	276 (13.39%)	
**Location/teaching status**					** < 0.001**
Rural	50,958 (14.73%)	50,587 (14.77%)	133 (10.64%)	238 (11.55%)	
Urban nonteaching	163,422 (47.25%)	161,923 (47.27%)	542 (43.36%)	957 (46.43%)	
Urban teaching	131,515 (38.02%)	130,074 (37.97%)	575 (46.00%)	866 (42.02%)	
**Hospital region**					** < 0.001**
Northeast	69,477 (20.00%)	68,815 (20.00%)	260 (20.68%)	402 (19.41%)	
Midwest	80,083 (23.05%)	79,432 (23.09%)	237 (18.85%)	414 (19.99%)	
South	135,271 (38.94%)	133,985 (38.95%)	451 (35.88%)	835 (40.32%)	
West	62,532 (18.00%)	61,803 (17.96%)	309 (24.58%)	420 (20.28%)	

Significant values are in bold

*THA* total hip arthroplasty, *ORIF* open reduction and internal fixation, *CRIF* close reduction and internal fixation

Patients’ postsurgical outcomes are shown in Table 2. Significant differences were found between the three groups in all outcomes of interest: in-hospital mortality, non-routine discharge, postoperative complications (i.e., acute kidney injury, AMI, pneumonia and respiratory complications, acute kidney injury, hemorrhagic complications, infection/sepsis, neurological complications and thrombocytopenia), extended LOS, and hospital costs (all *P* < 0.05) (Table 2).

**Table 2:** Outcomes of study population chronic liver disease status

Term	Total (*n* = 347,363)	No liver disease (*n* = 344,035)	Non-cirrhotic liver disease (*n* = 1257)	Cirrhosis (*n* = 2071)	*P*-value
**In-hospital mortality**					** < 0.001**
No	340,126 (97.92%)	336,928 (97.93%)	1228 (97.69%)	1970 (95.12%)	
Yes	7237 (2.08%)	7107 (2.07%)	29 (2.31%)	101 (4.88%)	
**Non-routine discharge**					** < 0.001**
No	34,070 (9.81%)	33,730 (9.80%)	135 (10.74%)	205 (9.90%)	
Yes	306,056 (88.11%)	303,198 (88.13%)	1093 (86.95%)	1765 (85.22%)	
Die in hospital	7237 (2.08%)	7107 (2.07%)	29 (2.31%)	101 (4.88%)	
**Extended LOS**^**a**^					** < 0.001**
No	249,667 (73.40%)	247,647 (73.50%)	808 (65.80%)	1212 (61.52%)	
Yes	90,459 (26.60%)	89,281 (26.50%)	420 (34.20%)	758 (38.48%)	
**Complication**					
Any complication	202,413 (58.27%)	200,049 (58.15%)	787 (62.61%)	1577 (76.15%)	** < 0.001**
Stroke / TIA	7619 (2.19%)	7548 (2.19%)	32 (2.55%)	39 (1.88%)	0.4367
Delirium	14,753 (4.25%)	14,602 (4.24%)	66 (5.25%)	85 (4.10%)	0.1996
AMI	11,003 (3.17%)	10,922 (3.17%)	40 (3.18%)	41 (1.98%)	0.0083
Pneumonia and respiratory complication	34,855 (10.03%)	34,463 (10.02%)	132 (10.50%)	260 (12.55%)	0.0006
Acute kidney injury	32,501 (9.36%)	31,981 (9.30%)	166 (13.21%)	354 (17.09%)	** < 0.001**
Hemorrhagic complication	134,416 (38.70%)	132,910 (38.63%)	480 (38.19%)	1026 (49.54%)	< 0.001
Infection / sepsis	34,345 (9.89%)	33,950 (9.87%)	154 (12.25%)	241 (11.64%)	0.0005
Neurological complication	6280 (1.81%)	6199 (1.80%)	28 (2.23%)	53 (2.56%)	0.0192
Thrombocytopenia	20,006 (5.76%)	19,136 (5.56%)	209 (16.63%)	661 (31.92%)	** < 0.001**
Digestive system complication	2146 (0.62%)	2116 (0.62%)	7 (0.56%)	23 (1.11%)	0.0157
Venous thromboembolic complication	2317 (0.67%)	2297 (0.67%)	7 (0.56%)	13 (0.63%)	0.8691
Wound / Technical complication	2626 (0.76%)	2599 (0.76%)	11 (0.88%)	16 (0.77%)	0.8840
**Hospital cost**	48,326.89 (38,480.98)	48,187.59 (38,247.92)	62,906.23 (55,350.91)	62,861.23 (56,398.31)	** < 0.001**

*LOS* length of hospital stay, *AMI* acute myocardial infarction

^a^exclude those died in hospital

^b^*P*-value used for Fisher’s exact test or chi-square test

### Associations between study outcomes and non-cirrhotic chronic liver disease

Results of univariate and multivariate regression analysis on associations between outcomes and liver disease status are summarized in Tables 3 and 4. In univariate analysis, patients with non-cirrhotic liver disease had higher risk of any complication (OR: 1.205, 95% CI: 1.750–1.351), acute kidney injury (OR: 1.485, 95% CI: 1.260–1.749), extended LOS (OR: 1.442, 95% CI: 1.281–1.623) and greater hospital costs (beta: 14,718.64, 95% CI: 12,551.60–16,885.68) than those without liver disease. After adjusting for confounders, non-cirrhotic chronic liver disease was significantly associated with non-routine discharge (OR: 1.247, 95% CI: 1.038–1.498), acute kidney injury (OR: 1.266, 95% CI: 1.039–1.541), extended LOS (OR: 1.285, 95% CI: 1.122–1.473) and total hospital costs (beta: 9173.42, 95% CI: 6925.9–11,420.95) as compared to no liver disease (Tables 3 and 4).

**Table 3:** Associations between in-hospital mortality, any complication, acute kidney injury and chronic liver disease status

	**In-hospital Mortality** OR (95% CI)	**Any Complication** OR (95% CI)	**Acute kidney injury** OR (95% CI)
**Univariate analysis**			
No liver disease	ref	ref	ref
Non-cirrhotic liver disease	1.120 (0.774–1.619)	**1.205 (1.75–1.351)**	**1.485 (1.260–1.749)**
Cirrhosis	**2.431 (1.987–2.973)**	**2.293 (2.072–2.537)**	**2.012 (1.793–2.257)**
**Multivariate analysis**			
No liver disease	ref	ref	ref
Non-cirrhotic liver disease	1.364 (0.914–2.034)	0.976 (0.849–1.122)	**1.266 (1.039–1.541)**
Cirrhosis	**2.325 (1.849–2.922)**	**1.295 (1.143–1.467)**	**1.242 (1.077–1.433)**

Significant values are shown in bold

Multivariate analysis after adjusting for significant baseline characteristics in Table 1

**Table 4:** Associations between non-routine discharge, extended LOS, hospital costs and chronic liver disease status

	**Non-routine discharge** OR (95% CI)	**Extended LOS** OR (95% CI)	**Hospital Cost** β (95% CI)
**Univariate analysis**			
No liver disease	ref	ref	Ref
Non-cirrhotic liver disease	0.936 (0.792–1.108)	**1.442 (1.281–1.623)**	**14,718.64 (12,551.60–16,885.68)**
Cirrhosis	**1.286 (1.117–1.481)**	**1.735 (1.584–1.901)**	**14,673.64 (12,994.98–16,352.30)**
**Multivariate analysis**			
No liver disease	ref	ref	ref
Non-cirrhotic liver disease	**1.247 (1.038–1.498)**	**1.285 (1.122–1.473)**	**9173.42 (6925.9,11,420.95)**
Cirrhosis	**1.650 (1.412–1.928)**	**1.405 (1.263–1.562)**	**6680.24 (4921.53,8438.95)**

*LOS* length of hospital stay

Significant values are shown in bold

Multivariate analysis after adjusting for significant baseline characteristics in Table 1

### Associations between study outcomes and cirrhosis

In univariate analysis, patients with cirrhosis were at higher risk of in-hospital mortality (OR: 2.431, 95%CI: 1.987–2.973), any complication (OR: 2.293, 295% CI; 2.072–2.537), acute kidney injury (OR: 2.012, 95% CI: 1.793–2.257), non-routine discharge (OR: 1.286, 95%CI: 1.117–1.481), extended LOS (OR: 1.735, 95% CI: 1.584–1.901) and total hospital costs (beta: 14,673.64, 95% CI: 12,994.98–16,352.30) than those without liver disease. After adjusting for confounders, cirrhosis remained significantly associated with higher risk of in-hospital mortality (OR: 2.325, 95% CI: 1.849–2.922), any complication (OR: 1.295, 95% CI: 1.143–1.467), acute kidney injury (OR: 1.242, 95% CI: 1.177–1.433), non-routine discharge (OR: 1.650, 95% CI: 1.412–1.928), extended LOS (OR: 1.405, 95% CI: 1.263–1.562) and hospital costs (6680.24, 95% CI: 4921.53–8438.95) compared to no liver disease. (Tables 3 and 4). Supplementary Table 2 displays variables analyzed in multivariate regression.

## Discussion

In the present study, assessment of the clinical characteristics and in-hospital outcomes of 347,363 older adult hip fracture patients undergoing emergent surgery revealed that liver cirrhosis doubles the risk of in-hospital death, and independently predicts worse postoperative outcomes. Specifically, the outcomes of patients with liver cirrhosis included more non-routine discharges, extended LOS, higher rates of any complication and acute kidney injury, and greater hospital costs. In addition, non-cirrhotic chronic liver disease was independently associated with higher risk of acute kidney injury, non-routine discharge, extended LOS and hospital costs, but did not predict increased in-hospital mortality or rate of any complication.

Multiple studies addressing the influence of cirrhosis/chronic liver disease on orthopedic surgeries, including elective hip and knee arthroplasty and spine surgery, had findings in line with those of the present study regarding poorer outcomes [13, 14, 20, 21]. In a cohort of 693,610 patients undergoing orthopedic surgery, mainly hip/knee arthroplasty and spinal laminectomy/fusion, those with cirrhosis generally had greater post-operative mortality, longer hospital stays and higher costs [13]. In a review study, authors concluded that comorbid chronic liver disease was prevalent in patients undergoing total hip arthroplasty, leading to increased mortality and perioperative complications, including infection, instability, perioperative fracture and revision surgery [14]. Analysis of data from the National Surgical Quality Improvement Program (2006–2015) of the American College of Surgeons revealed that, in over 20,000 patients undergoing surgery for degenerative cervical spine disease, liver disease independently predicted poorer 30-day outcomes [20].

Only a few studies explored the role of liver disease specifically in hip fracture surgeries. Chen et al. [21] evaluated post-fracture outcomes in 688,290 hospitalized hip fracture patients over age 20 in the Taiwan National Health Insurance database, finding that patients with cirrhosis had higher risk of fracture, more complications and higher 30-day mortality than other fracture patients. Authors emphasized careful patient selection and preoperative evaluation of patients’ liver function. Chang et al.[9] analyzed data of 117,129 hip fracture patients, including 4048 with cirrhosis, from the Taiwan National Health Insurance database 2004–2010, finding that cirrhotic patients were relatively younger, with higher incidence of hip fracture, higher postoperative infection rates, and 2–3 times higher mortality rates at 3 years post-op. Grau et al. [22] examined complications and non-routine discharge rates in surgically-treated hip fracture patients, identifying a negative association between non-cirrhotic HCV infection and outcomes, with higher risk of both perioperative complications and non-routine discharge to extended care facilities. A significant association was found between age and in-hospital mortality after fall-related hip fracture in older adults in a study conducted in Spain, and mortality was influenced by comorbidities, including congestive heart failure, metastatic cancer, pulmonary function disorders, peripheral vascular disease and liver disease [23]. Assessment of mortality risk in hip fracture patients using prospective data from the Danish registries found that 30-day mortality was markedly increased in liver disease patients, especially those with liver cirrhosis, compared to those without liver disease [24].

Discrepancies in results between these studies and ours may stem from differences in study population, such as broader age range, types of liver disease or fractures. For example, while some studies focused only on the influence of cirrhosis, and others on hepatitis C (HCV) infection [21], the present study evaluated the influence of both cirrhosis and chronic non-cirrhotic liver disease and analyzed them separately. Montomoli et al. [24] used a design most similar to ours, obtaining data from the Danish National Health Service with an analytic sample of 152,180 hip fracture patients, but only reported mortality data and did not adjust for various hospital-related factors as in the present study. And unlike in the present study, pathologic fractures were not excluded in that analysis, making the outcomes prone to bias.

Acute kidney injury is a frequent complication following hip fracture. Hong et al. [25] found that acute kidney injury predicted both in-hospital and long-term mortality in older adults undergoing hip fracture surgery. A 2020 study also reported that acute kidney injury was associated with longer admissions and increased mortality following geriatric hip fracture surgery; preexisting renal disease, heart disease and postoperative blood transfusion were identified as independent risk factors for developing post-surgical acute kidney injury [26]. Findings of the present study indicate that both non-cirrhotic chronic liver disease and cirrhosis increase the risk of acute kidney injury following hip fracture surgery during admission, and this may partially explain the extended LOS and excess mortality found.

### Strengths and limitations

The main strength of the present study is the use of the large inpatient sample of the comprehensive NIS database, which closely represents the US population. Inclusion and exclusion criteria were also strictly set to ensure uniform distribution of patients’ baseline characteristics. In addition, confounders either at patient and hospital levels were carefully controlled in regression analysis. To date, the present study is the most comprehensive analysis of this topic, including data of both cirrhotic and non-cirrhotic cohorts and considering a broad range of postoperative morbidities.

Several limitations must be noted, primarily due to the use of administrative claims data, which may underestimate the prevalence of chronic liver disease [24]. In addition, the total number of patients with non-cirrhotic liver disease was lower than that of cirrhosis in the present study, suggesting that the patients with non-cirrhotic liver disease were underestimated. The possibility that some elderly patients might be misclassified into the group of no liver disease in this study cannot be ruled out. Elderly patients often had multiple comorbidities. When the principal diagnosis for a particular hospital admission was not liver-related, chronic liver disease might not be documented on the discharge record in the NIS database, even though the particular elderly patient had comorbid chronic liver disease. Moreover, chronic hepatitis patients without an acute exacerbation during admission might not be not coded in the NIS database, and therefore were not captured by this study. Undiagnosis/undetection of chronic liver disease could also resulted in the underestimated incidence of chronic liver disease. In addition, the NIS data do not provide the stage of cirrhosis or information about medications prescribed and intraoperative variables such as length of operation or laboratory parameters, so these could not be analyzed in the present study. The NIS database provides only inpatient data with no follow-up data after discharge, which precluded evaluating late morbidities and mortality. Further long-term study is needed to expand the analyses and confirm results of the present study.

## Conclusions

Non-cirrhotic chronic liver disease and cirrhosis independently predict non-routine discharge, acute kidney injury, prolonged LOS and greater hospital costs among geriatric hip fracture patients undergoing surgical repair. In addition, cirrhosis significantly increases the risk of in-hospital mortality and rate of any postoperative complications. These findings suggest that vigilance and careful monitoring of older patients with liver disease are highly warranted.

## Supplementary Information

**Additional file 1: Table S1.** Codes for defining liver disease and postoperative complications. **Table S2.** Multivariate analysis of study variables.

## Data Availability

The datasets generated and/or analysed during the current study are available in the NIS Database. HCUP. 2005–2014. Agency for Healthcare Research and Quality, Rockville, MD. https://www.hcup-us.ahrq.gov/db/nation/nis/nisdbdocumentation.jsp.
