# Supplementary material for: Chronic liver disease and cirrhosis increase morbidity in geriatric patients treated surgically for hip fractures: analysis of the US Nationwide Inpatient Sample
Source: BMC Geriatr. 2022 Feb 23;22:150. doi: 10.1186/s12877-022-02832-y (PMC8867787; doi:10.1186/s12877-022-02832-y)
Supplement: Supplementary file 1 — Additional file 1: Table S1. Codes for defining liver disease and postoperative complications. Table S2. Multivariate analysis of study variables. [file 12877_2022_2832_MOESM1_ESM.docx]

**Chronic liver disease and cirrhosis increase morbidity in geriatric patients treated surgically for hip fractures: Analysis of the US Nationwide Inpatient Sample**

**Journal:** Archives of Orthopaedic and Trauma Surgery

Feng-Jen Tseng, MD^1,2,^, Guo-Hau Gou, Ph.D ^3^, Sheng-Hao Wang, MD^3,4^, Jia-Fwu Shyu, Ph.D^5^, Ru-Yu Pan, Ph.D^3,4*^

^1^ Department of Orthopedics, Hualien Armed Forces General Hospital, Hualien 971, Taiwan, ROC

^2^ Department of Life Science and the institute of Biotechnology, National Dong Hwa University, Hualien 974 ,Taiwan, ROC

^3^ Graduate Institute of Medical Sciences, National Defense Medical Center, Taipei, 11490, Taiwan, ROC

^4^ Department of Orthopaedics, Tri-Service General Hospital, National Defense Medical Center, Taipei, 11490, Taiwan, ROC

^5^ Department of Biology and Anatomy, National Defense Medical Center, Taipei, 11490, Taiwan, ROC

***Correspondence to:**

Ru-Yu Pan, Ph.D

Graduate Institute of Medical Sciences, National Defense Medical Center

**Address:** No. 161, Section 6, Mingchuan E. Road, Neihu District 114,Taipei,Taiwan,ROC

**Tel:** 886-2-87923100

**E-mail:** [ru-yu123@hotmail.com](mailto:ru-yu123@hotmail.com)

| **Supplementary Table 1. Codes for defining liver disease and postoperative complications.** | |
| --- | --- |
| Disease | Code |
| ***Liver disease*** |  |
| Non-cirrhotic liver disease | Chronic hepatitis B (070.21–070.33), chronic hepatitis C (070.41, 070.44, 070.54, 070.7), unspecified chronic hepatitis (571.40, 571.41, 571.49), autoimmune hepatitis (571.42), primary sclerosing cholangitis (576.1), alcoholic liver disease (571.3, 571.0), nonalcoholic fatty liver disease (571.8), unspecified chronic liver disease (571.9) |
| Cirrhosis | Alcoholic cirrhosis of liver (571.2), Cirrhosis of liver without mention of alcohol (571.5), Biliary cirrhosis (571.6) |
| ***Complication*** |  |
| Stroke / TIA | 997.02, 433-435 |
| Delirium | 292.81, 293, 293.0, 293.1, 293.8, 293.81–84, 293.89, 293.9, 780.97 |
| AMI | 410, 410.01, 410.11, 410.2, 410.21, 410.3, 410.31, 410.4, 410.41, 410.5, 410.51, 410.6, 410.9, 410.91, 997.1 |
| Pneumonia and respiratory complication | 480-480.9, 481, 482-482.9, 483, 483.1, 483.8, 484, 484.1, 484.3, 484.5-8, 485, 486, 487, 507, V12.61, 997.3, 122CCS, 512, 514, 518.4, 518.5, 518.7, 518.81, 518.82, 518.84 |
| Acute kidney injury | 584.5-584.9, 157CCS, V45.1 |
| Hemorrhagic complication | 998.1, 719.15, V58.2, Procedure code 99.0 |
| Infectious/sepsis | 595.0, 599.0, 997.5, 996.31, 996.64, V13.02, 682.6, 686.9, 891, 894, 996.62,996.64, 996.66-7, 996.69, 998.5, 998.59, 999.31, 86.04, 86.09, 86.22, 86.28, 86.3, 995.9, 320, 041, 790.7, 038, 519.2, 785.52, 999.3, 998.5 |
| Neurological complication | 345, 997.01, 997.09 |
| Thrombocytopenia | 287.4, 287.5 |
| Digestive system complication | 997.4 |
| Venous thromboembolic | 415.x, 451.11, 451.19, 451.81-84, 451.89, 451.9, 451.2, 453.x, 997.2 |
| Wound/ Technical complication | 998.3, 998.30, 998.31, 998.32, 998.33, 998.6, 998.83, 998.51, 996.1, 996.74, 998.2, 998.4, 998.7, 998.8, 998.81, 998.89, 998.9 |
| TIA, transient ischemic attack; AMI, acute myocardial infarction. | |

| **Supplementary Table 2. Multivariate analysis of study variables.** | | | | | | |
| --- | --- | --- | --- | --- | --- | --- |
|  | **In-hospital mortality** | **Non-routine discharge** | **Extended LOS ^a^** | **Any complication** | **Acute kidney injury** | **Hospital cost** |
|  | aOR (95% CI) | aOR (95% CI) | aOR (95% CI) | aOR (95% CI) | aOR (95% CI) | β (95% CI) |
| **Liver disease** |  |  |  |  |  |  |
| No liver disease | Ref | Ref | Ref | Ref | Ref | Ref |
| Non-cirrhotic liver disease | 1.364 (0.914-2.034) | **1.247 (1.038-1.498)** | **1.285 (1.122-1.473)** | 0.976 (0.849-1.122) | **1.266 (1.039-1.541)** | **9173.42 (6925.9,11420.95)** |
| Cirrhosis | **2.325 (1.849-2.922)** | **1.650 (1.412-1.928)** | **1.405 (1.263-1.562)** | **1.295 (1.143-1.467)** | **1.242 (1.077-1.433)** | **6680.24 (4921.53,8438.95)** |
| **Age** |  |  |  |  |  |  |
| 65-74 | Ref | Ref | Ref | Ref | Ref | Ref |
| 75-84 | **1.84 (1.247-1.536)** | **2.102 (2.038-2.168)** | 1.014 (0.986-1.042) | **1.252 (1.222-1.283)** | **1.088 (1.041-1.138)** | **-1808.75 (-2226.21,-1391.3)** |
| 85+ | **2.132 (1.927-2.358)** | **3.168 (3.065-3.275)** | **0.971 (0.945-0.999)** | **1.558 (1.520-1.597)** | **1.245 (1.191-1.302)** | **-3227.39 (-3650.57,-2804.21)** |
| **Gender** |  |  |  |  |  |  |
| Female | Ref | Ref | Ref | Ref | Ref | Ref |
| Male | **1.657 (1.566-1.753)** | **0.967 (0.941-0.994)** | **1.354 (1.326-1.382)** | - | **1.502 (1.457-1.547)** | **3878.63 (3555.63,4201.62)** |
| **Procedure** |  |  |  |  |  |  |
| Hemiarthroplasty | Ref | Ref | Ref | Ref | Ref | Ref |
| THA | - | **0.675 (0.628-0.725)** | 1.047 (0.987-1.109) | **1.095 (1.037-1.156)** | 1.009 (0.916-1.112) | **10509.03 (9583.34,11434.72)** |
| ORIF | - | **0.605 (0.581-0.630)** | **0.689 (0.667-0.711)** | **0.687 (0.668-0.708)** | **0.836 (0.795-0.880)** | **-14107.08 (-14598.36,-13615.8)** |
| CRIF | - | **0.609 (0.584-0.635)** | **0.546 (0.528-0.565)** | **0.649 (0.630-0.669)** | **0.848 (0.805-0.894)** | **-14332.9 (-14839.24,-13826.55)** |
| Internal fixation alone | - | **0.534 (0.508-0.561)** | **0.547 (0.524-0.570)** | **0.541 (0.522-0.561)** | **0.806 (0.756-0.860)** | **-16221.64 (-16833.31,-15609.96)** |
| **Fracture type** |  |  |  |  |  |  |
| Cervical | Ref | Ref | Ref | Ref | Ref | Ref |
| Peri/Intertrochanteric | **1.120 (1.057-1.188)** | **1.700 (1.643-1.759)** | **1.325 (1.287-1.364)** | **2.055 (2.004-2.107)** | **1.262 (1.206-1.320)** | **7394.9 (6968.14,7821.66)** |
| Subtrochanteric | **1.296 (1.169-1.438)** | **1.844 (1.74-1.46)** | **1.588 (1.523-1.656)** | **3.131 (3.011-3.256)** | **1.530 (1.436-1.631)** | **14017.23 (13373.74,14660.71)** |
| **Income** |  |  |  |  |  |  |
| Lowest quartile | Ref | Ref | Ref | Ref | Ref | Ref |
| Second quartile | 0.977 (0.907-1.053) | 0.996 (0.964-1.029) | **0.927 (0.904-0.951)** | 1.003 (0.980-1.026) | **0.955 (0.918-0.993)** | **-790.2 (-1177.04,-403.36)** |
| Third quartile | **0.892 (0.825-0.965)** | **0.949 (0.917-0.9820** | **0.917 (0.894-0.942)** | 1.005 (0.982-1.029) | 0.978 (0.940-1.018) | 143.84 (-258.7,546.36) |
| Fourth quartile | **0.880 (0.812-0.955)** | **0.927 (0.894-0.961)** | **0.934 (0.909-0.959)** | **1.093 (1.066-1.121)** | **0.952 (0.913-0.993)** | **2501.9 (2080.79,2923.01)** |
| **Race/ethnicity** |  |  |  |  |  |  |
| White | Ref | Ref | Ref | Ref | Ref | Ref |
| Black | 0.898 (0.766-1.054) | **0.842 (0.789-0.898)** | **1.505 (1.436-1.578)** | **1.243 (1.185-1.303)** | **1.487 (1.393-1.587)** | **6463.7 (5678.52,7248.87)** |
| Hispanic | 1.024 (0.896-1.170) | **0.698 (0.663-0.735)** | **1.355 (1.301-1.412)** | 1.023 (0.984-1.064) | **1.146 (1.076-1.220)** | **13674.31 (13014.75,14333.87)** |
| Asian | 1.038 (0.812-1.327) | **0.713 (0.653-0.778)** | **1.470 (1.367-1.581)** | 1.031 (0.962-1.106) | **1.230 (1.104-1.369)** | **6211.19 (5031.96,7390.42)** |
| Others | 1.009 (0.843-1.208) | **0.768 (0.716-0.825)** | - | **1.158 (1.098-1.222)** | **1.113 (1.017-1.216)** | **4925.45 (4022.61,5828.29)** |
| **Insurance status / Primary Payer** | |  |  |  |  |  |
| Medicare | Ref | Ref | Ref | Ref | Ref | Ref |
| Medicaid | 0.969 (0.708-1.325) | **0.256 (0.234-0.280)** | **1.993 (1.829-2.172)** | 1.014 (0.931-1.106) | 1.142 (0.993-1.312) | **7126.51 (5668.09,8584.93)** |
| Private | 0.980 (0.866-1.109) | **0.732 (0.699-0.766)** | **1.200 (1.155-1.247)** | 0.971 (0.93-1.006) | 1.026 (0.965-1.090) | **618.58 (22.81,1214.35)** |
| Self / no charge / others | 1.021 (0.802-1.299) | **0.349 (0.324-0.375)** | **1.386 (1.291-1.489)** | 0.976 (0.913-1.044) | 0.995 (0.882-1.122) | **-1291.51 (-2430.01, -153.01)** |
| **Comorbidities** |  |  |  |  |  |  |
| Anemia | - | **1.040 (1.013-1.067)** | **1.324 (1.299-1.349)** | **1.649 (1.620-1.679)** | **1.112 (1.080-1.144)** | **2669.15 (2369.64,2968.65)** |
| Rheumatoid arthritis/collagen vascular diseases | 0.852 (0.719-1.009) | 1.040 (0.976-1.109) | **0.901 (0.856-0.948)** | **1.053 (1.007-1.101)** | **0.877 (0.808-0.952)** | **-781.32 (-1539.34, -23.3)** |
| Congestive heart failure | **2.332 (2.202-2.470)** | **1.483 (1.429-1.538)** | **1.921 (1.877-1.965)** | **1.469 (1.434-1.504)** | **1.694 (1.641-1.750)** | **7142.32 (6756.17,7528.48)** |
| Chronic pulmonary disease | **1.427 (1.344-1.515)** | **1.200 (1.164-1.237)** | **1.340 (1.311-1.370)** | **1.187 (1.163-1.212)** | 0.974 (0.942-1.008) | **3655.14 (3306.64,4003.63)** |
| Coagulopathy | 1.082 (0.990-1.182) | **1.122 (1.069-1.179)** | **1.222 (1.182-1.264)** | **24.675 (22.661-26.867)** | **1.497 (1.432-1.566)** | **8320.97 (7772.71,8869.23)** |
| Depression | **0.782 (0.714-0.856)** | **1.192 (1.150-1.229)** | **0.953 (0.928-0.979)** | **1.142 (1.115-1.170)** | 0.964 (0.924-1.005) | -324.61 (-733.93,84.72) |
| Diabetes | **-** | **1.193 (1.158-1.229)** | **1.111 (1.087-1.135)** | 1.104 (1.082-1.127) | **1.240 (1.201-1.281)** | **1545.24 (1201.39,1889.1)** |
| Hypertension | **0.666 (0.629-0.705)** | **1.034 (1.007-1.061)** | - | **0.993 (0.976-1.011)** | 1.033 (0.998-1.068) | -216.93 (-524.5,90.65) |
| Hypothyroidism | **0.868 (0.809-0.931)** | **1.033 (1.002-1.065)** | **0.936 (0.914-0.957)** | **1.044 (1.023-1.066)** | **1.041 (1.005-1.077)** | **539.36 (193.83,884.89)** |
| Fluid/electrolyte disorders | **1.797 (1.703-1.896)** | **1.226 (1.193-1.259)** | **1.781 (1.747-1.815)** | **1.634 (1.604-1.664)** | **2.871 (2.793-2.952)** | **7739.93 (7437.24,8042.63)** |
| Other neurological disorders | - | **1.230 (1.192-1.270)** | **1.149 (1.123-1.175)** | 1.425 (1.395-1.455) | **0.905 (0.872-0.940)** | 93.91 (-262.39,450.2) |
| Obesity | **0.693 (0.575-0.836)** | **1.307 (1.218-1.403)** | **1.242 (1.180-1.307)** | **1.044 (0.993-1.096)** | **1.444 (1.347-1.549** | **6881.51 (6050.52,7712.49)** |
| Peripheral vascular disorders | **1.287 (1.182-1.403)** | **1.113 (1.0306-1.494)** | **1.178 (1.139-1.218)** | **1.207 (1.168-1.247)** | **1.048 (0.999-1.098)** | **2694.84 (2152.95,3236.74)** |
| Psychoses | - | **1.397 (1.306-1.494)** | **1.394 (1.331-1.460)** | **1.134 (1.084-1.186)** | **0.877 (0.811-0.949)** | **4103.37 (3339.25,4867.49)** |
| Pulmonary circulation disorders | **1.658 (1.519-1.808)** | **1.371 (1.286-1.462)** | **1.569 (1.506-1.634)** | **1.579 (1.509-1.652)** | **1321 (1.249-1.397)** | **8865.97 (8183.06,9548.87)** |
| Renal failure | **1.711 (1.606-1.822)** | **1.327 (1.277-1.378)** | **1.222 (1.192-1.254)** | **1.960 (1.909-2.013)** | **7.190 (6.979-7.407)** | **5683.81 (5265.76,6101.86)** |
| Valvular disease | 0.968 (0.901-1.040) | **1.057 (1.015-1.101)** | **1.211 (1.178-1.244)** | **1.142 (1.112-1.174)** | 0.988 (0.949-1.029) | **1092.32 (649.01,1535.64)** |
| Weight loss | **2.382 (2.199-2.582)** | **1.487 (1.401-1.579)** | **2.236 (2.152-2.324)** | **1.693 (1.623-1.766)** | **1.705 (1.619-1.795)** | **21427.68 (20777.49,22077.87)** |
| Cardiac dysrhythmias | **2.346 (2.220-2.480)** | **1.430 (1.389-1.473)** | **1.841 (1.806-1.878)** | 1.291 (1.267-1.316) | **1.173 (1.138-1.209)** | **8169.58 (7849.37,8489.78)** |
| Tobacco use | **0.683 (0.628-0.743)** | **0.867 (0.840-0.896)** | **0.765 (0.745-0.785)** | **0.916 (0.895-0.938)** | **0.911 (0.875-0.948)** | 137.24 (-259.42,533.88) |
| **Hospital bed size** |  |  |  |  |  |  |
| Large (>450) | Ref | Ref | Ref | Ref | Ref | Ref |
| Medium (250-450) | - | **0.936 (0.911-0.961)** | **0.897 (0.879-0.916)** | **1.089 (1.068-1.109)** | **0.918 (0.890-0.948)** | **-2691.49 (-3007.62,-2375.36)** |
| Small (<250) | - | **0.881 (0.850-0.912)** | **0.754 (0.734-0.776)** | **1.120 (1.093-1.148)** | **0.825 (0.790-0.861)** | **-6099.42 (-6515.25,-5683.59)** |
| **Location/teaching status** |  |  |  |  |  |  |
| Rural | Ref | Ref | Ref | Ref | Ref | Ref |
| Urban nonteaching | **0.899 (0.826-0.978)** | **1.353 (1.303-1.403)** | 1.004 (0.975-1.034) | **0.861 (0.839-0.884)** | **0.349 (1.283-1.417)** | **11969.52 (11527.42,12411.61)** |
| Urban teaching | 0.931 (0.855-1.014) | **1.225 (1.180-1.272)** | **1.119 (1.086-1.153)** | **0.855 (0.832-0.878)** | **1.645 (1.565-1.730)** | **13940.64 (13486.16,14395.13)** |
| **Hospital region** |  |  |  |  |  |  |
| Northeast | Ref | Ref | Ref | Ref | Ref | Ref |
| Midwest | **0.863 (0.794-0.937)** | **0.703 (0.676-0.730)** | **0.569 (0.553-0.586)** | **0.668 (0.651-0.685)** | **0.925 (0.885-0.967)** | **-6915.41 (-7351.34,-6479.48)** |
| South | **0.883 (0.824-0.948)** | **0.744 (0.720-0.769)** | **0.789 (0.771-0.808)** | **0.883 (0.863-0.902)** | 1.012 (0.975-1.051) | 223.99 (-146.88,594.85) |
| West | **0.75 (0.693-0.826)** | **0.624 (0.601-0.648)** | **0.546 (0.531-0.562)** | **0.793 (0.773-0.814)** | **0.880 (0.841-0.920)** | **14552.57 (14105.48,14999.66)** |

Significant values are shown in bold.

THA, total hip arthroplasty; ORIF, open reduction and internal fixation; CRIF, close reduction and internal fixation.
